# Energy-Efficient Self-Organized Coverage Control in LoRaWAN Inspired by Satellite Behavior of Japanese Tree Frogs

**DOI:** 10.3390/s26196153

**Published:** 2026-09-28

**Authors:** Daichi Kominami, Yushi Hosokawa, Ikkyu Aihara, Masayuki Murata

**Affiliations:** 1 Graduate School of Information Science and Technology, The University of Osaka, Osaka 565-0871, Japan; 2 Faculty of Engineering, Information and Systems, University of Tsukuba, Ibaraki 305-8577, Japan

**Keywords:** LPWA network, self-organized control, biomimetics, communication

## Abstract

The Long-Range Wide-Area Network (LoRaWAN) is one of the leading low-power wide-area network specifications owing to its capabilities for long-range communication and energy savings. For large-scale sensing applications by a large number of LoRa nodes, it is important to improve communication performance and energy saving. However, redundant sensing and transmissions consume node energy, while simultaneous transmissions, particularly from hidden nodes, cause packet collisions. Centralized optimization of these problems requires the collection of network-wide information and may impose substantial communication overhead due to its narrow communication bandwidth. In this paper, we propose a distributed method for jointly controlling sensing coverage, node energy consumption, and transmission timing using locally exchanged information. Our main idea is to learn from the swarm intelligence of organisms that perform efficient reproductive behavior. The proposed method extends a previously developed mathematical model that reproduced the chorus and satellite behavior observed in three Japanese tree frogs. Whereas the original model describes the satellite behavior of a frog relative to a nearby caller, the proposed method generalizes this interaction to multiple wireless nodes associated with the same sensing target. By embedding target-point and node-state information in transmitted packets, each node identifies the *k*th-ranked node associated with the target and autonomously determines whether to remain active or enter a low-power satellite state. This mechanism regulates the time- and target-averaged number of active sensing nodes toward *k* without collecting global node-distribution information. We further introduce an in-phase-flag mechanism that modifies node-specific phase interactions to suppress persistent packet collisions between hidden nodes located two hops apart. Simulation results show that the proposed method reduces transmission energy consumption by 65% for average 1-coverage and by 46% for average 2-coverage compared with the method without satellite-state control. In the collision evaluation, the two-hop packet collision rate was 9.36% without phase control and 4.49% with the basic phase-control mechanism. By additionally applying the in-phase-flag-based hidden-node collision-control mechanism, the two-hop collision rate was further reduced to 3.51%, while maintaining a low one-hop collision rate. These results demonstrate that the proposed extension of the frog-behavior model can jointly regulate sensing redundancy and suppress data collisions through distributed local interactions.

## 1. Introduction

Low-power wide-area network (LPWAN) technology enables low-power communication over a wide area and is considered a key technology for the Internet of Things (IoT) [1]. The Long-Range Wide-Area Network (LoRaWAN) is one of the most widely deployed LPWAN specifications using unlicensed frequency bands [2,3]. LoRaWAN can provide long-range communication with low power consumption. However, transmission errors occur because of signal attenuation and packet collisions [4]. These errors can be reduced by appropriately assigning spreading factors (SFs), transmission powers, and frequency channels to LoRa nodes.

Various methods have been proposed to improve the communication reliability, scalability, and energy efficiency of LoRaWAN. The Adaptive Data Rate (ADR) mechanism adjusts the data rate, SF, and transmission power according to the observed link condition. Finnegan et al. analyzed the convergence behavior of ADR and proposed an improved method that reduces the time required for end devices to reach appropriate data rates [5]. Other studies have proposed contention-aware data-rate assignment [6] and joint allocation of SFs and transmission powers considering co-SF and inter-SF interference [7].

Resource allocation and transmission scheduling have also been studied to reduce packet collisions in dense LoRaWANs. The Collision Avoidance Resource Allocation (CARA) method assigns channels, SFs, transmission powers, and code rates to improve network capacity and reduce packet collisions [8]. Garrido-Hidalgo et al. proposed a multi-agent method that determines slot lengths and node allocation for collision-free communication in large-scale multi-SF LoRaWANs [9]. These methods explicitly allocate communication resources or transmission opportunities to individual nodes. Energy efficiency has also been addressed through joint optimization of communication parameters in other wireless network settings. For example, Zhu et al. jointly considered routing and power assignment to reduce energy consumption in end-to-end retransmission systems [10], while energy-aware communication control based on deep reinforcement learning has been investigated for resource-constrained sensor networks [11].

Distributed collision avoidance is another approach. Gamage et al. proposed LMAC, which uses LoRa channel activity detection to implement carrier-sense multiple access [12]. Carrier sensing enables a node to postpone its transmission when the channel is busy. However, it cannot always avoid collisions between hidden nodes that cannot directly detect each other. Schedule-based methods can avoid such collisions by assigning transmission opportunities, but they require synchronization and schedule information. Synchronization-based transmission has also been investigated in sensor networks; for example, Wang proposed a synchronous transmission method based on resonance technology [13].

The existing methods mainly improve communication performance by adjusting radio parameters, allocating communication resources, or scheduling transmissions. They do not generally consider sensing redundancy. When several nodes can observe the same target point, not all of them need to remain active and transmit sensing data simultaneously. In this study, we control the number of active sensing nodes as well as their transmission timing. Our objective is to maintain an average coverage level close to a required value *k* while reducing energy consumption and packet collisions. To achieve this objective without collecting global information, each node needs to make decisions based on information obtained from nearby nodes.

Recently, increasing attention has been focused on biomimetics, which is the creation of new technologies inspired by the behavior of living organisms [14]. The cooperative behavior that emerges from the autonomous behavior of individuals in a group is called swarm intelligence [15]. Individuals exhibiting swarm intelligence make behavioral decisions using information obtained from a limited range. Therefore, they do not require global knowledge of the entire group. This property provides scalability with respect to the population size and adaptability to environmental changes. Many studies have applied swarm intelligence to various engineering problems, such as network control [16], path planning [17] and task offloading [18].

In this paper, we apply swarm intelligence to the control of an LPWAN that provides sustainable sensing services while maintaining a required level of coverage. Among organisms exhibiting swarm intelligence, we focus on Japanese tree frogs (Figure 1). We have previously modeled their chorus and satellite behavior mathematically [19] and applied their chorus behavior to autonomous network control [20,21].

In many frog species, males produce successive calls to attract females. A male frog produces these calls by expanding and contracting its vocal sac. The call of one male induces other males to call, resulting in a group chorus. This calling behavior is effective for attracting females but consumes a large amount of energy.

The chorus of Japanese tree frogs has an interesting characteristic. It exhibits anti-phase synchronization on a short time scale and synchronization on a long time scale. We have previously applied these characteristics to autonomous and energy-efficient communication control [20,21]. In this study, we also focus on the satellite behavior of male frogs, which is another characteristic of their reproductive behavior.

Satellite behavior is a strategy in which a male remains silent near another calling male and attempts to intercept a female attracted to the caller [22]. Calling males consume energy to attract females, whereas satellite males reduce their energy consumption by remaining silent. At the individual level, these behaviors are selfish reproductive strategies. However, a combination of chorus and satellite behaviors can improve the energy efficiency of the whole group.

Our previous study proposed a hybrid dynamical model in which frogs autonomously switch between chorus, sleep, and satellite states through excitatory and inhibitory interactions [19]. We showed that the model could reproduce both collective chorus and satellite behavior observed in a group of three Japanese tree frogs. The simulation results also showed that satellite males could delay the energy depletion of the whole group while dividing the maximum chorus activity into two levels over the chorusing period. These results provided an ecological understanding of how autonomous state transitions among individual frogs affect the chorus activity and energy consumption of the whole group.

In this study, we focus on the engineering application of this swarm intelligence. We use the previously developed chorus and satellite behavior model as the basis for a new distributed control algorithm for wireless sensing networks. In the biological model, each frog autonomously changes its behavioral state based on interactions with nearby frogs. By mapping these behavioral states and local interactions to the operating states and information exchanges of wireless nodes, we aim to control sensing coverage and energy consumption without collecting global network information.

We assume a LoRaWAN that collects sensing data, such as temperature, humidity, and sound, which are strongly related to the locations of the sensing nodes. When several nodes can observe the same target point, only some of them need to perform sensing and transmit the resulting data. We therefore use the frog-inspired state-transition model to control the number of active sensing nodes. Nodes that are not required for sensing enter the satellite state and reduce their communication activity.

The biological model considers the satellite behavior of a frog relative to a nearby calling frog. In contrast, a wireless sensing network may require *k* active nodes around each observation target, as illustrated in Figure 2. We extend the state-transition model so that the time-averaged number of active nodes around each target approaches *k*. Unlike frogs, wireless nodes can include explicit information in their transmitted packets. In the proposed method, each node transmits information about its observation target and residual energy. Each node then uses the *k*th-ranked residual-energy value among the nodes associated with the same target to determine whether it remains active or enters the satellite state. In addition, we introduce asymmetric thresholds for transitions into and out of the satellite state to suppress frequent state changes.

Our previous frog-inspired network methods used the calling interactions and anti-phase synchronization of frogs for energy-efficient communication and autonomous transmission-timing control [20,21]. However, those methods did not consider a required sensing coverage level or the selection of redundant sensing nodes based on satellite behavior. They also did not consider persistent packet collisions between hidden nodes. In the present study, we combine average *k*-coverage control based on satellite behavior with an extended phase-interaction method for hidden-node collision avoidance.

The technical contributions of this study are summarized as follows. First, we extend the chorus and satellite behavior model developed for a small group of frogs to distributed average *k*-coverage control in LoRaWAN. Second, we exploit the ability of wireless packets to carry information about observation targets and residual energy, and introduce a *k*th-rank-based state-transition rule and asymmetric transition thresholds. Third, we extend the one-hop anti-phase interaction model by introducing an in-phase flag mechanism that suppresses persistent in-phase synchronization between nodes that cannot directly exchange control information but are connected through a common neighboring node. Such node pairs can still transmit to the same gateway in a conventional single-gateway LoRaWAN and may therefore cause repeated overlapping uplink transmissions. Through computer simulations, we evaluate the average sensing coverage, energy consumption, and packet collisions for directly connected node pairs and such indirectly connected two-hop node pairs.

The remainder of this paper is organized as follows. In Section 2, we explain a model in which the timing of each individual’s vocalization is represented by a phase oscillator model. In Section 3, we describe a mathematical model for the chorus and satellite behavior of Japanese tree frogs. In Section 4, we explain how to apply the model to LoRa nodes. In Section 5, we show the results of performance evaluation by computer simulation. Finally, in Section 6, we summarize this study and discuss future research issues.

## 2. Mathematical Model of Frog Vocalization

In this section, we explain how the timing of frog vocalizations is represented by a phase oscillator model and is determined based on interactions with other frogs. The further description of this model is explained in Ref. [21].

In the model, each frog has one oscillator. The oscillator is a variable that increases with angular velocity ω and returns to 0 when it exceeds 2π. When the oscillator reaches θ=0, the owner frog of the oscillator vocalizes. At this time, if the phase difference between the frogs within interactive range of each other is longer than the period of each call, a collision in vocalization can be avoided.

The oscillator possessed by each frog interacts with the oscillators possessed by interactive frogs through the vocalization. The frog that listens to the vocalization of another frog updates the phase of its own oscillator based on the phase difference with its own oscillator according to the Equations (1) and (2). This avoids the situation where frogs adjacent to each other vocalize at the same time.

Here, in-phase synchronization of oscillators of frogs that are not adjacent to each other cannot be avoided. However, there is a possibility of *communication collisions* due to the hidden node problem in wireless communication when we apply this model to wireless communications. Therefore, we propose a method that uses Equation (3), which is a modification of Equation (2), as the interaction function.

### 2.1. Phase Interaction Function

The phase of the oscillator at each frog varies according to dθ/dt=ω as long as there is no interaction with other nodes. Here, ω is a positive constant. When data or beacons are received from neighboring nodes, the phase is updated according to the following equation.

(1) $$\theta_{n}\left(t\right)\leftarrow \Gamma_{n,m}+\theta_{n}\left(t\right)$$

For $\Gamma_{n,m}$, we use the interaction function (Equation (2)).

(2) $$\Gamma_{n,m}\left(\theta_{n}-\theta_{m}\right)=\kappa \left(1-\frac{2}{1+\exp(\alpha \left(\pi -\left(\theta_{n}-\theta_{m}\right)\right))}\right)$$

Equation (2) approaches a value of 0 the closer the phase difference between itself and the oscillator of the adjacent node is to π. Therefore, it prevents oscillator synchronization between adjacent frogs, but it does not avoid in-phase synchronization of oscillators of frogs that are not interactive with each other. The hidden node problem that arises in wireless communications is the problem of radio collisions caused by two transmitting nodes simultaneously transmitting data to a receiving node located in between them. In our preliminary simulations, in wireless networks where the phase of nodes is updated with Equations (1) and (2), collisions can occur whenever the hidden node problem arises in the steady state. Therefore, we propose a method using Equation (3), which is a modified version of Equation (2), as the interaction function.

(3) $$\Gamma_{n,m}\left(\theta_{n}-\theta_{m}\right)=\kappa \left(1-\frac{2}{1+\exp(\alpha \left(\pi -\left(\theta_{n}-\theta_{m}\right)+S\right))}\right)$$

In Equation (3), we add a parameter *S* to Equation (2). When S=0, Equation (3) is equal to Equation (2). The addition of *S* shifts the stability point from π by *S*. Each frog (node) can set the value of *S* individually and the initial value is S=0. The value of *S* is updated by instructions from a common neighboring node of the two nodes whose oscillators synchronize to avoid in-phase synchronization. Details are explained in the next section.

Figure 3 shows each interaction function when each parameter of the interaction term is set to κ=0.05, α=4.0, and *S* is set to −0.6π, 0, and 0.6π.

### 2.2. Application of Frog Vocalization Model to Wireless Communication

As well as the frog vocalization model, each wireless node has one oscillator. When data needs to be transmitted, each node waits until the next time when the phase of its own oscillator reaches θ=0 before transmitting. At this time, if the phase difference between the nodes within communication range of each other (neighboring nodes) is longer than the time required for data transmission, a collision can be avoided. If the node does not have data at the time when the phase of its own oscillator is θ=0, it transmits a small frame (beacon).

In order to avoid a situation in which hidden nodes are in-phase synchronized with each other, a node will notify the in-phase flag to one of the nodes when the phase difference between adjacent nodes is less than a threshold value (set to 0.05π in this paper). Here, the node detecting the in-phase synchronization is a common neighboring node that can receive transmissions from both hidden nodes. The reason why only one node is notified of the in-phase flag is that if two or more nodes update *S* in the same way, there will be no difference in the change from the stability point between the hidden nodes and in-phase synchronization cannot be avoided. One node is selected randomly, and as long as the selected node is the target to be notified of the in-phase flag, the notification is continued for that node.

The node receiving the in-phase flag cannot determine whether in-phase synchronization with the hidden node is occurring regularly or temporarily. Therefore, the node that receives the in-phase flag changes its own *S* value if the in-phase flag is sent from the same node for more than the threshold number of consecutive times. The reason why the node sending the in-phase flag continuously notifies the same node is because it is necessary for this conditional judgment. In addition, to determine that the in-phase flags are consecutive, the method of judging whether or not they were received within a certain period of time from the previous in-phase flag is used. After satisfying the above conditions, the in-phase flag is sent, and the node that receives the in-phase flag for itself randomly changes the parameter *S* in its own interaction function.

The implementation of this neighbor-to-neighbor control signaling in the LoRaWAN architecture is described in Section 4.

## 3. Mathematical Model of Frog Chorus Behavior

First, we explain the mathematical model, proposed in [19], that reproduces the chorus and satellite behavior of frogs. In this mathematical model, each frog is assumed to have three states: “chorus state,” “sleep state,” and “satellite state.” In the chorus state, a frog is calling to attract female frogs. In the sleep state, a frog waits for recovery from fatigue without calling. In the satellite state, a frog suppresses its energy consumption without calling. The state of a frog *n* is denoted by $s_{n}$ and here $s_{n}=0$ represents the chorus state, $s_{n}=1$ represents the sleep state, and $s_{n}=2$ represents the satellite state. The state transitions are probabilistically made at regular intervals based on Equations (10)–(13). To determine the state transition probability, two variables representing fatigue and physical strength are used. We denote the degree of fatigue for frog *n* as $T_{n}$ and the physical strength as $E_{n}$.

Chorus state

The vocalization of a frog in the chorus state is modeled by the phase oscillator model. In this model, the timing of the vocalization of frog *n* is determined by the phase $\theta_{n}$. In the chorus state, $\theta_{n}$ repeatedly increases from 0 to 2π, and when $\theta_{n}=2\pi$, frog *n* vocalizes and $\theta_{n}$ is reset to 0. The frog also changes its own phase by the vocalization of frogs other than itself [Equation (4)]. The phase is not changed in the case of sleep or satellite states.

Sleep state

The continuous contraction and expansion of the vocal sac causes the frog to produce sounds. This behavior is considered to accumulate physical fatigue, which is expressed by the fatigue level $T_{n}$. The fatigue level increases with each vocalization and recovers when the frog is in a sleep or satellite state. The transition probability from the chorus state to the sleep state (or vice versa) is determined by the value of $T_{n}$.

Satellite state

Frogs lose weight after a night of choral singing owing to the consumption of their physical energy. As an alternative mating strategy, male frogs sometimes stay silent in the vicinity of another caller to intercept a female attracted, which is known as satellite behavior and allows males to reduce energy consumption. The physical strength ($E_{n}$) decreases with each vocalization, and in this model, it is assumed that no state includes recovery.

### 3.1. State Transition Model

In the model of [19], the transition between the satellite and chorus states is not direct, and only the transitions between the chorus state and the sleep state and between the sleep state and the satellite state are considered. First, we describe the time variation of the phases that change fatigue and physical strength, and then we describe the probabilistic state transition.

#### 3.1.1. Time Variation of Phase

$\theta_{n}$, $E_{n}$, and $T_{n}$ change in each of the chorus, sleep, and satellite states as follows:

- Chorus state:
  (4) $$\begin{array}{ccc} \frac{d\theta_{n}}{dt} & = & \omega +\sum_{m\in Nb(n)}\delta \left(\theta_{m}\right)\Gamma_{nm}\left(\theta_{n}-\theta_{m}\right), \end{array}$$
  (5) $$\begin{array}{ccc} \frac{dE_{n}}{dt} & = & -\delta (\theta_{n}), \end{array}$$
  (6) $$\begin{array}{ccc} \frac{dT_{n}}{dt} & = & +\delta (\theta_{n}). \end{array}$$
- Sleep and satellite states:
  (7) $$\begin{array}{ccc} \frac{d\theta_{n}}{dt} & = & 0, \end{array}$$
  (8) $$\begin{array}{ccc} \frac{dE_{n}}{dt} & = & 0, \end{array}$$
  (9) $$\begin{array}{ccc} \frac{dT_{n}}{dt} & = & -\alpha_{T}. \end{array}$$

In these state equations, *N* is the total number of individuals and Nb(n) is the set of individuals whose distance from *N* is within the threshold $r_{0}$ and are in the chorus state, and $\delta (\theta_{n})$ is a delta function defined in [19], whereby for each vocalization, the energy $E_{n}$ decreases by 1 and the fatigue $T_{n}$ increases by 1. $E_{n}$ and $T_{n}$ ($0\leq E_{n}\leq E_{max}$ and $0\leq T_{n}\leq T_{max}$, respectively) have upper and lower bounds that are never exceeded.

#### 3.1.2. Probabilistic State Transition

In [19], the timing of individual vocalizations was modeled deterministically and the state transitions were modeled probabilistically based on the results observed experimentally. For an individual *n*, the probability of transition from the chorus state to the sleep state (denoted by $P_{n}^{call\to silent}$), from the sleep state to the chorus state (denoted by $P_{n}^{silent\to call}$), and from the sleep state to the satellite state (denoted by $P_{n}^{silent\to satellite}$), and the transition probability from the satellite state to the sleep state (denoted by $P_{n}^{satellite\to silent}$) are represented by the following equations, respectively:

(10) $$\begin{array}{ccc} P_{n}^{call\to silent} & = & G_{1}\left(T_{n}\right), \end{array}$$

(11) $$\begin{array}{ccc} P_{n}^{silent\to call} & = & G_{2}\left(T_{n}\right)H\left(n\right), \end{array}$$

(12) $$\begin{array}{ccc} P_{n}^{silent\to satellite} & = & I_{1}\left(F_{n}\right), \end{array}$$

(13) $$\begin{array}{ccc} P_{n}^{satellite\to silent} & = & I_{2}\left(F_{n}\right). \end{array}$$

The transition from the chorus state to the sleep state is modeled based on their hypothesis that once a frog begins to call, it continues to vocalize as long as its fatigue level is small, and that it probabilistically transitions to a sleep state when its fatigue level becomes large. This modeling is represented by $G_{1}\left(T_{n}\right)$ in the following Equation (14):

(14) $$\begin{array}{ccc} G_{1}\left(T_{n}\right) & = & \frac{1}{\exp(-\gamma \left(T_{n}-\Delta T\right))+1}. \end{array}$$

$G_{1}\left(T_{n}\right)$ is a sigmoid function, which means that the probability of transitioning from the chorus state to the sleep state increases rapidly when the fatigue level becomes larger than a certain threshold value.

The transition from the sleep state to the chorus state is modeled based on the following hypothesis. When the degree of fatigue $T_{n}$ is small, the frog tends to start calling. In addition, as seen in many species, including the Japanese tree frog, when one individual starts calling, individuals in the vicinity tend to follow it and also start croaking. These are modeled by $G_{2}$ in Equation (15) and *H* in Equation (16).

(15) $$\begin{array}{ccc} G_{2}\left(T_{n}\right) & = & \frac{1}{\exp(\gamma \left(T_{n}-\left(T_{max}-\Delta T\right)\right))+1} \end{array}$$

(16) $$\begin{array}{ccc} H(n) & = & \left\{\begin{array}{cc} p_{low} & (\mathrm{if}\;s_{j}=0\;\;\exists j\in Nb\left(n\right)),\\ p_{high} & (otherwise). \end{array}\right. \end{array}$$

$G_{2}\left(T_{n}\right)$ is a sigmoid function, and when the fatigue level becomes smaller than a certain threshold value, the probability of transitioning from the sleep state to the chorus state increases rapidly. H(n) is a binary function, and the value is determined by whether other nearby individuals are vocalizing. *H* returns $p_{high}$ if there is at least one neighbor individual in the chorus state, and $p_{low}$ otherwise.

Finally, we describe the transition model from the sleep state to the satellite state (also from the satellite state to the sleep state). Here, the modeling is based on the hypothesis that the frog compares itself with its neighbors and transitions to the satellite state when it judges that a neighboring individual is more likely to attract female frogs. As a criterion for determining whether an individual is more likely to attract females, the model assumes that individuals that vocalize for a longer period of time are more likely to attract females. The number of consecutive vocalizations of frog *n* in the last chorus state, denoted by $F_{n}$, is used for this criterion.

When deciding whether to remain silent (satellite state), frog *n* compares its $F_{n}$ to *F* in its nearest neighbor. Note that the values of *F* of the neighbor are stored for a certain period of time. The transition probability from the sleep state to the satellite state, $I_{1}\left(F_{n}\right)$, and the transition probability from the satellite state to the sleep state, $I_{2}\left(F_{n}\right)$, are defined in Equation (18) and Equation (19), respectively.

(17) $$\begin{array}{ccc} F_{\max}\left(n\right) & = & \max_{j\in Nb^{sate}\left(n\right)\;\;s_{j}=0}F_{j}, \end{array}$$

(18) $$\begin{array}{ccc} I_{1}\left(F_{n}\right) & = & \frac{1}{\exp(-\epsilon \left(F_{\max}\left(n\right)-F_{n}-\lambda \right))+1}, \end{array}$$

(19) $$\begin{array}{ccc} I_{2}\left(F_{n}\right) & = & \frac{1}{\exp(\epsilon \left(F_{\max}\left(n\right)-F_{n}+\lambda \right))+1}, \end{array}$$

where $Nb^{sate}\left(n\right)$ is the set of individuals whose distance from frog *n* is less than or equal to $r_{0}^{sate}$. λ is an offset that sets the threshold on the attractiveness gap ($F_{\max}\left(n\right)-F_{n}$) at which a node changes its state.

## 4. Proposed Coverage Control Method

We describe the average *k*-coverage design method for LoRaWAN based on the model of frog behavior described in the previous section. Note that ‘average coverage’ represents the time-averaged number of active nodes that can observe each target point. The proposed method regulates this value toward *k*, but does not guarantee that every target point is covered by at least *k* active nodes at every instant.

In applying the proposed control model to LoRaWAN, we distinguish the communication of sensing data from the exchange of control information among neighboring nodes. Sensing data are transmitted to the gateway through the conventional LoRaWAN data plane. In contrast, the beacon and in-phase-flag signaling described in Section 2.2 is assumed to be exchanged through a peer-to-peer control plane between directly reachable nodes. This control plane may, for example, be implemented by direct communication using the LoRa physical layer independently of the standard LoRaWAN MAC procedure.

First, we explain how to map frog behavior to LoRaWAN node behavior. The state of the *n*th LoRa sensor node is represented as $s_{n}$, just like an individual frog. The chorus state ($s_{n}=0$) corresponds to an active communication state, whereas the sleep state ($s_{n}=1$) and satellite state ($s_{n}=2$) correspond to low-activity states in which sensing-data transmission is reduced or suspended. These states determine the activity of the LoRaWAN data plane, while the local control signaling required for state transitions is handled separately through the peer-to-peer control plane.

In the mathematical model of frog behavior, listening to other individuals’ vocalizations does not consume physical energy, but in wireless communication, the antenna needs to be ready to receive data transmitted by other nodes, and this consumes power. Therefore, even when $s_{n}=1,\;2$, nodes periodically activate the control interface for a short period ($\frac{2\pi }{\omega }$) to exchange control information with neighboring node. Outside these period, the radio interface can remain inactive to reduce energy consumption.

The interpretation in LoRaWAN of the variables in each state of the frog model is shown in Table 1.

*T* is a variable that represents the degree of muscle fatigue of the frog as it makes its vocal sacs resonate, and is used to model frogs’ inability to vocalize continuously for long periods of time. By using the functions $G_{1}$ and $G_{2}$ with *T* as an argument, the length of time for continuous vocalization and the length of time for sleeping and recovery from fatigue can be adjusted. In wireless communication, laws and wireless standards may not allow continuous communication for more than a certain amount of time. Using *T* makes it possible to operate with such restrictions. In addition, it is possible to determine the time for communication and the time to sleep to satisfy the operating time required by the application by designing $G_{1}$ and $G_{2}$.

The transition probabilities between $s_{n}=0,\;1,\;2$ are calculated based on Equation (10). Note that in a LoRaWAN, the right-hand side of Equation (7) is set to ω, and the phase is assumed to be constantly updated even in the sleep and satellite states. Then, every time $\theta_{n}=0$ in all states, a probabilistic decision for state transition is made. In the frog model, the number of vocalizations ($F_{n}$) was used as an indicator of the attractiveness of an individual, but wireless nodes can directly exchange information representing their attractiveness through the control plane. In this study, the physical strength value ($E_{n}$) is included in the control information exchanged among neighboring nodes.

To achieve average *k*-coverage, information about the observation target point associated with each node is also required. Therefore, we assume that all nodes know their own locations via Global Positioning System (GPS). In this study, node mobility is not considered, and therefore the node position does not need to be updated frequently. We assume that the position is acquired only during initialization or at sufficiently long intervals, and the energy consumed for positioning is not included in the present evaluation. If GPS is unavailable, other localization methods based on network connectivity, received signal strength, or externally configured position information may be used instead, as long as each node can determine the observation target points associated with its location.

Based on their locations, each node determines the target points that it can observe. Each node then includes the identifier of its selected observation target in the control information exchanged with neighboring nodes. Nodes that can observe multiple target points select one of them randomly.

If we apply the frog model directly, the node with the highest attractiveness has a higher probability of not being in the satellite state, and the other nodes have a higher probability of being in the satellite state. Then, the probabilistic state transition achieves 1-coverage on average. When there are more than *K* nodes near the target point (K>k), average *k*-coverage can be achieved by replacing the highest attractiveness with the *k*th highest attractiveness in the right-hand side of Equation (17).

In addition, Equations (18) and (19) use a common variable λ for the function $I_{1}$, which represents the ease of transition from the sleep state to the satellite state, and $I_{2}$, which represents the ease of transition from the satellite state to the sleep state. A sleep node moves to the satellite state when a neighbor is more attractive by more than $\lambda_{\mathrm{in}}$, whereas a satellite node returns to the sleep state only when it becomes more attractive than its neighbors by more than $\lambda_{\mathrm{out}}$. Because we set $\lambda_{\mathrm{in}}\ll \lambda_{\mathrm{out}}$, the two thresholds form an asymmetric hysteresis band in which neither transition is triggered; this suppresses rapid oscillation between states and stabilizes the set of satellite nodes, which is essential for maintaining average *k*-coverage.

The parameters used by the proposed control method are maintained locally at each node. When network-side reconfiguration is required, such parameters may also be updated through scheduled LoRaWAN downlinks. For example, Class B provides periodic ping slots synchronized by network beacons, allowing the network server to deliver configuration information to end devices without requiring continuous reception.

## 5. Simulation Results

In this section, we show that in the proposed method, LoRa nodes can achieve average *k*-coverage by autonomous decisions without using global node distribution information. An example of node distribution in our simulation is shown in Figure 4, where an area 2000 m square is divided into 3×3 subareas, and the center of each subarea is set as the observation target point. Forty nodes were placed at random locations, with the guarantee that at least three nodes are always included in each subarea. The communication range of the nodes was 1000 m, and the sensing range of information at the observation point was 500 m. The initial value of *T* was set to 0, and the physical strength *E* was randomly set between 4600 and 5000. The phase oscillator period was set to 4 s (ω=π/2 rad/s). The size of the data packet is 128 bytes and it is sent once every 30 phase oscillator cycles (every 120 s) at a transmission rate of 10 kbps. Also the size of the beacon packet is set to 16 byte. The other parameters of the phase oscillator model and the state transition model are summarized in Table 2. Note that, basically, the radio antenna of each node is ready to receive only when necessary. Therefore, its energy reduces at the same timing as the transmission of other nodes. For simplicity, in the following simulations, we assume that power is reduced only by transmission.

Figure 5 and Figure 6 show the results of the state transitions and the power consumption of each node when there is no state transition to the satellite state. The vertical axis of Figure 5 is the percentage of node states, and the horizontal axis is the simulation time. It shows that the chorus state and the sleep state are periodically repeated. This indicates that the chorus is synchronous, with vocalizations starting at the same time and pausing as the individuals become fatigued. This result is the same as the results of a previous study [21]. In the Figure 6, the vertical axis is residual energy, which decreases in a staircase fashion with time. This result is also the same as the results of our previous studies.

Here, we show the results when nodes apply the satellite state. Figure 7 shows the percentage of nodes in each state, and Figure 8 shows the average coverage, which is the average number of non-satellite nodes in the nine subareas. It is worth noting that while nodes change their state based on the locally available information, the ratio of the satellite state is almost constant in Figure 7a. In addition, the non-satellite nodes periodically repeat the chorus and sleep state, as in the case that does not consider the satellite state (Figure 5). Because node-state transitions are stochastic, they are not always the same, but in Figure 8, when the parameters for $I_{1}$ and $I_{2}$ are set for 1-coverage, the average coverage value is almost 1 in Figure 8a; also, the average coverage value is almost 2 in Figure 8b.

Due to the satellite state, nodes that are not needed for average *k*-coverage consume less power, as shown in Figure 9. Of course, the smaller the value of *K*, the fewer the number of nodes to be activated at the same time, and thus the higher the power-saving effect. The same simulation was performed 10 times, changing the initial placement of nodes, the power consumption is successfully reduced by 65% (46%) in the case of average 1-coverage (2-coverage) shown in Figure 9 compared with the case without considering satellite behavior.

In the simulation, data collisions occur when the timing of transmission overlaps with nodes one or two hops away. Figure 10 illustrates the timing of collision occurrence. It shows the percentage of the number of nodes in each state and the timing when the collision occurred.

The original frog vocalization model avoids synchronization with the individuals one hop ahead, but the proposed method also avoids synchronization with the individuals two hops ahead. Although collision occurs at the timing of chorus initiation, we confirmed that collision is avoided over time when the proposed method is used. This is because the probability of collision avoidance increases as the anti-phase synchronization progresses after the start of the chorus.

We evaluated data packet collisions based on the transmission timing of all active nodes. A transmission was counted as a collision when there existed at least one other transmission whose start time was within 0.1024 s (8×128byte/10kbps), corresponding to the packet transmission duration. Collision events were separately classified according to whether the corresponding transmitting nodes were one-hop pairs or two-hop pairs. Each transmission was counted at most once for each collision, even when it overlapped with multiple transmissions. Note that the first 1000 s of each simulation were excluded from the collision statistics. During this period, all nodes remained in the chorus state, and the state-control mechanism had not yet reduced the number of simultaneously active nodes. We therefore evaluated collisions only after 1000 s, where the proposed state-control mechanism was operating under its normal conditions.

As a reference, we first evaluated a simplified case without any phase control. One active node was randomly selected from each of the 3×3 subareas, resulting in nine active nodes. Their initial phases were independently assigned at random and then fixed throughout each trial. The evaluation was repeated for 10,000 trials, with 30,000 transmissions generated in each trial. The resulting mean one-hop and two-hop collision rates were 6.59% and 9.36%, respectively.

We next evaluated the phase-control mechanism without the additional mechanism for avoiding in-phase synchronization between hidden nodes. In this case, 90 one-hop collisions and 717 exact two-hop collisions were observed among 15,984 transmissions, corresponding to collision rates of 0.56% and 4.49%, respectively.

Then, we examined the sensitivity of the additional hidden-node collision-control mechanism to the conditions used for determining consecutive in-phase flags. Once an in-phase relation is detected, in-phase flags are generally received consecutively from the same node. Since such in-phase relations in the present system often appear only transiently, we focused on relatively small parameter values in order to evaluate the effect of reaction speed. Specifically, the required number of consecutive in-phase flags was set between 1, 3, and 5, while the allowable interval between successive flags was set among 1, 3, and 5 oscillator cycles.

Table 3 summarizes the results. When the required number of consecutive flags was set to 1, the two-hop collision rate decreased to 3.51%, irrespective of the allowable interval. In contrast, waiting for multiple consecutive flags did not always improve the collision rate. For example, requiring three consecutive flags with an allowable interval of one oscillator cycle resulted in a two-hop collision rate of 7.16%. These results suggest that rapid response to transient in-phase synchronization is important in the present model. The results for allowable intervals of three and five oscillator cycles were identical for each threshold setting, indicating that extending the allowable interval beyond three cycles had little additional effect under the present conditions.

In the above evaluation, nodes repeatedly alternate between the chorus state and the sleep or satellite state, and hence the effective interaction graph changes frequently over time. Under such dynamic conditions, choosing a large $N_{f}$, which implicitly assumes that the phase relationship persists sufficiently long to approach a steady state, provides only limited benefit. A longer continuous chorus period can in principle be obtained by adjusting the fatigue-related parameters of the frog-behavior model, allowing more time for the oscillator dynamics to evolve before the network topology changes. There is also a fundamental limit on the number of simultaneously active nodes that can be accommodated by phase separation. In the present setting, one oscillator cycle is 4 s and the transmission duration of a 128-byte packet at 10 kbps is 0.1024 s; therefore, even under ideal phase allocation, at most approximately 39 transmissions can be placed within one oscillator cycle without temporal overlap. The collision rates shown here represent a conservative case in which every active node is assumed to transmit once in every oscillator cycle. In practical sensing applications, however, the probability of data generation per cycle is expected to be lower than one, and thus a larger number of nodes can potentially be accommodated. Accordingly, an appropriate target value of average *k*-coverage should be selected by jointly considering the chorus duration, packet transmission time, and expected data-generation rate.

## 6. Conclusions

LoRaWAN is a significant technology for realizing IoT and future mobile communication systems. In this study, we applied a mathematical model mimicking a Japanese tree frog chorus and its satellite behavior to a LoRaWAN to achieve autonomous *k*-coverage control on average. The simulation results show that the lifetime of a LoRaWAN can be extended while maintaining a certain degree of coverage. In addition, by applying the mathematical model to a LoRaWAN, we show that the collision avoidance due to anti-phase synchronization of frogs model over a short time scale can be achieved.

The present study has several limitations. First, the evaluation assumes static sensor nodes and does not explicitly account for the energy consumption required for positioning. Since node mobility is not considered in the present study, position information can be obtained only at initialization or updated infrequently. However, when the proposed method is applied to dynamic environments involving node mobility, the energy consumption associated with periodic position updates should be explicitly considered. Second, the peer-to-peer control signaling required by the proposed distributed algorithm is modeled abstractly, and detailed LoRa-PHY/MAC implementation issues are not evaluated. Third, the wireless channel and collision model used in the simulations is simplified and does not fully capture propagation loss, interference, capture effects, and other characteristics of practical LoRaWAN deployments. Future work will address these limitations through prototype implementation and evaluation under more realistic wireless and energy-consumption models.

## Figures and Tables

**Figure 1:**
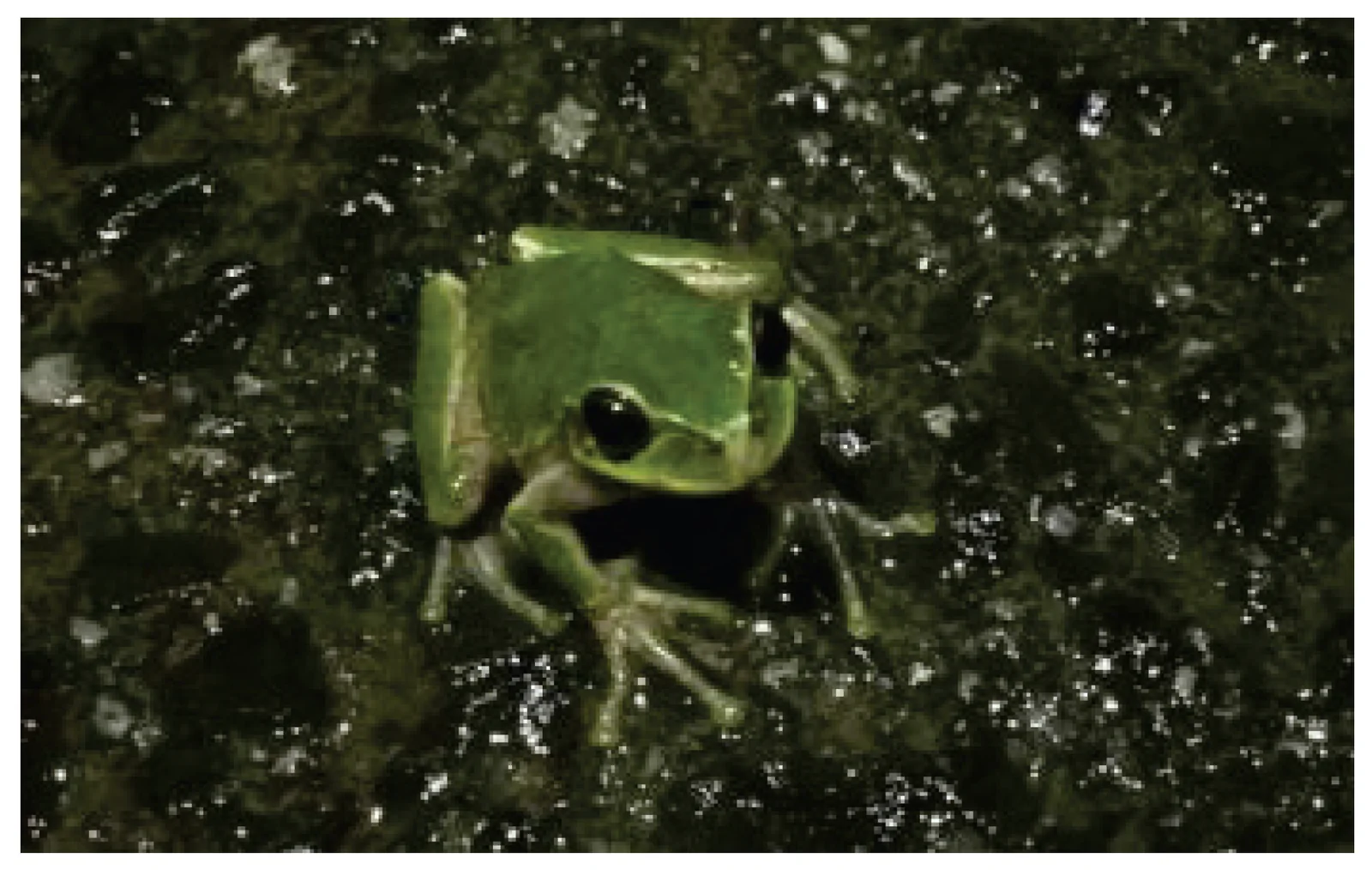
Japanese tree frog.

**Figure 2:**
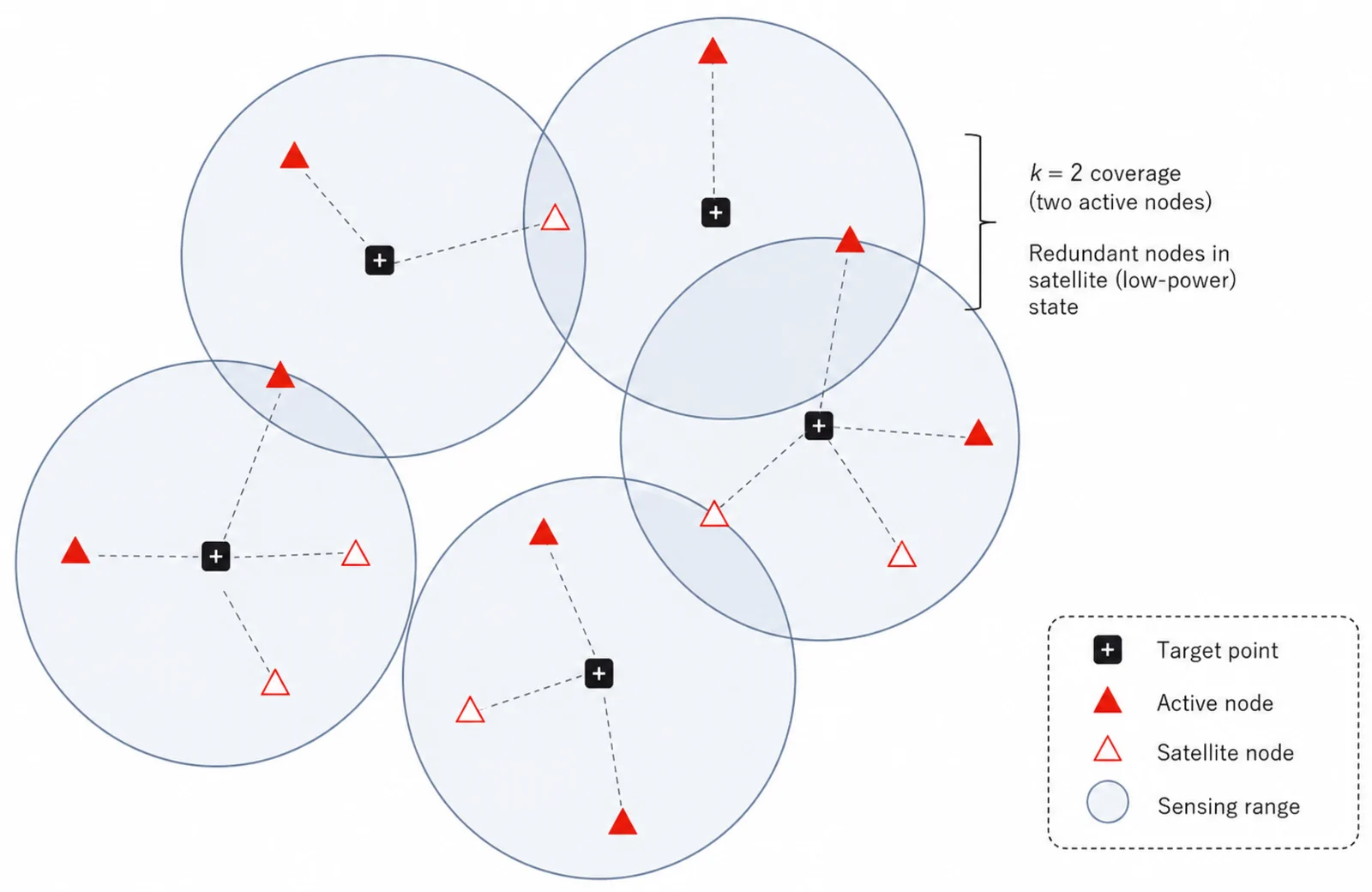
Two-coverage (the radius of the circle is the sensing range of the node).

**Figure 3:**
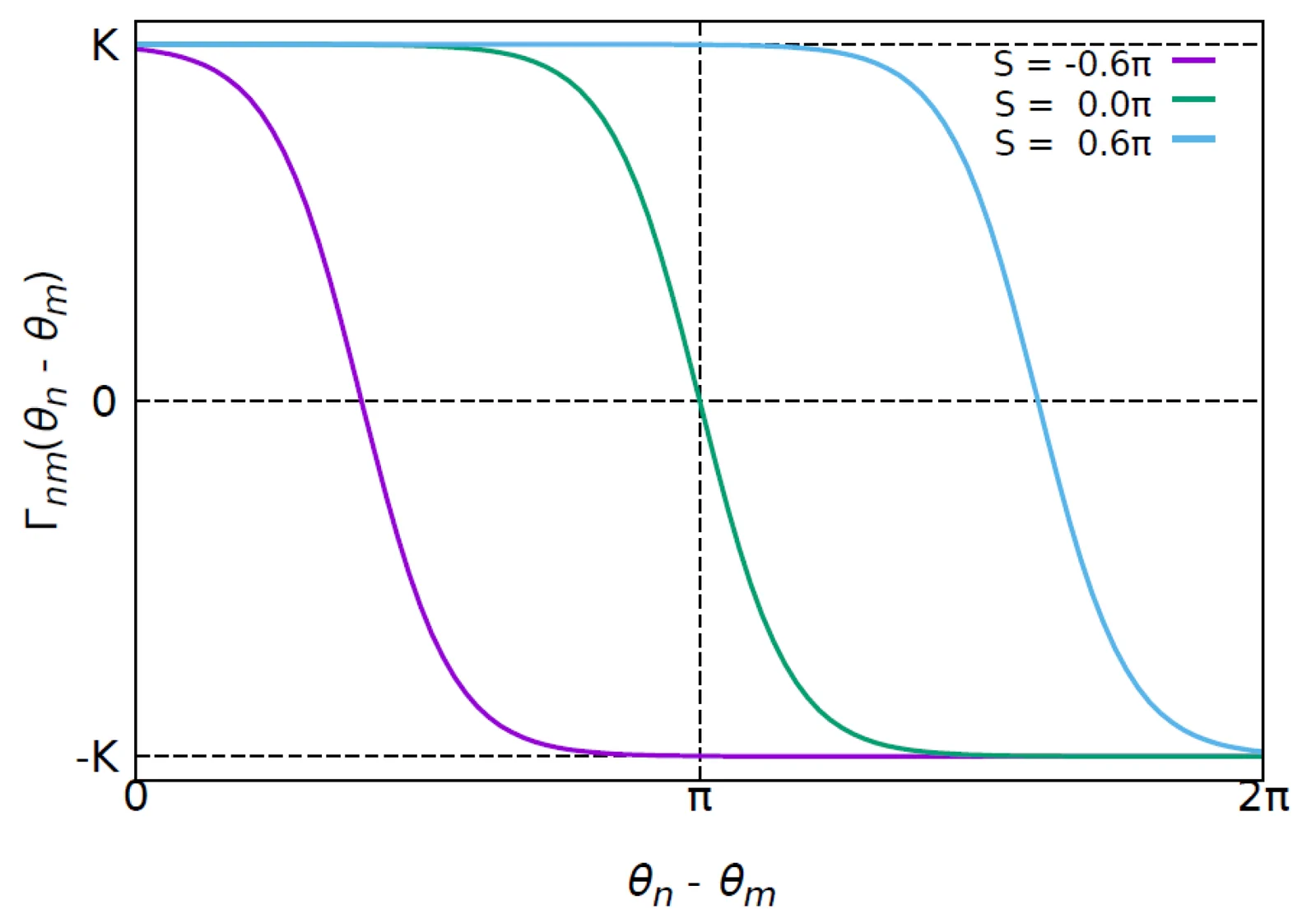
Phase interaction model.

**Figure 4:**
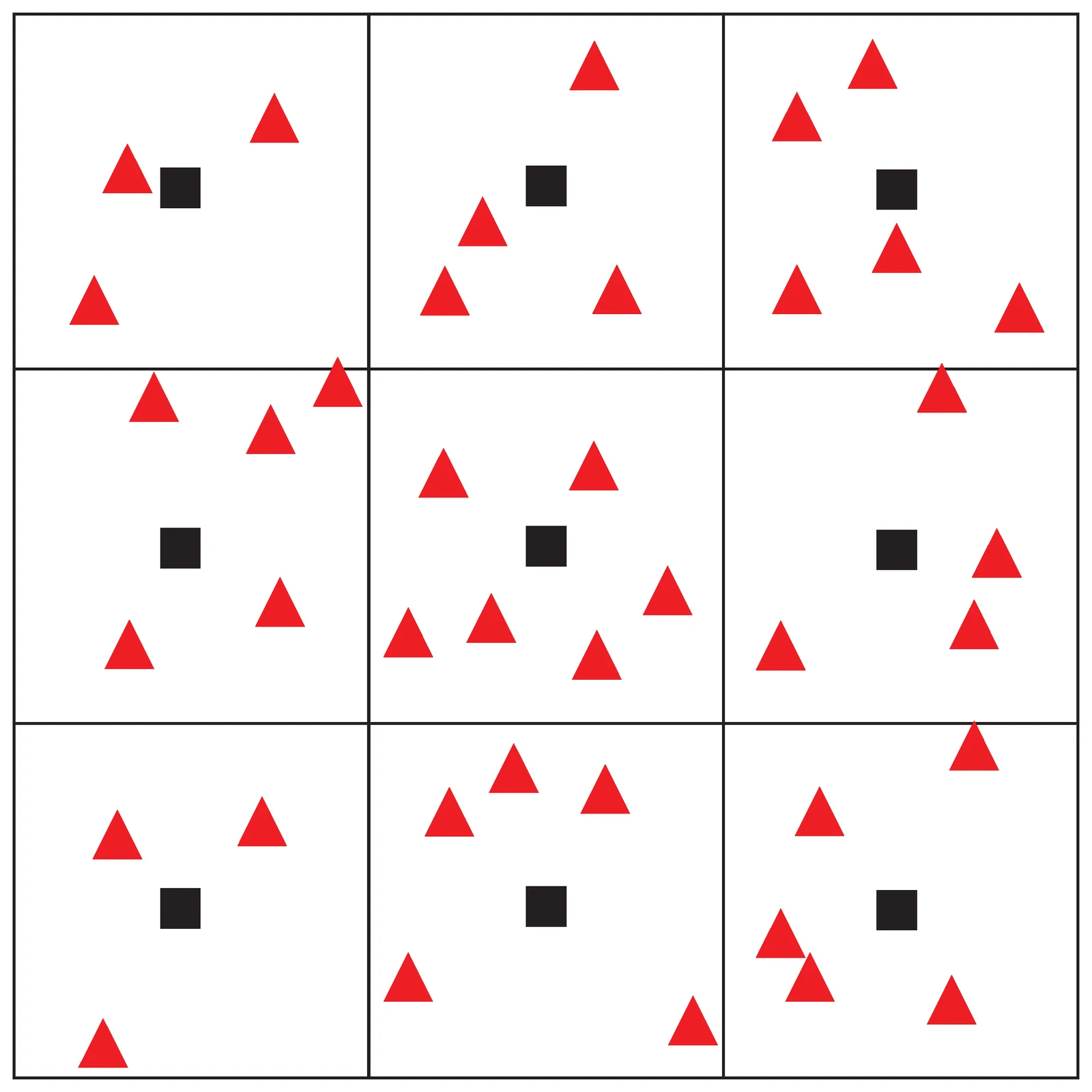
Topology (square = target point; triangle = node).

**Figure 5:**
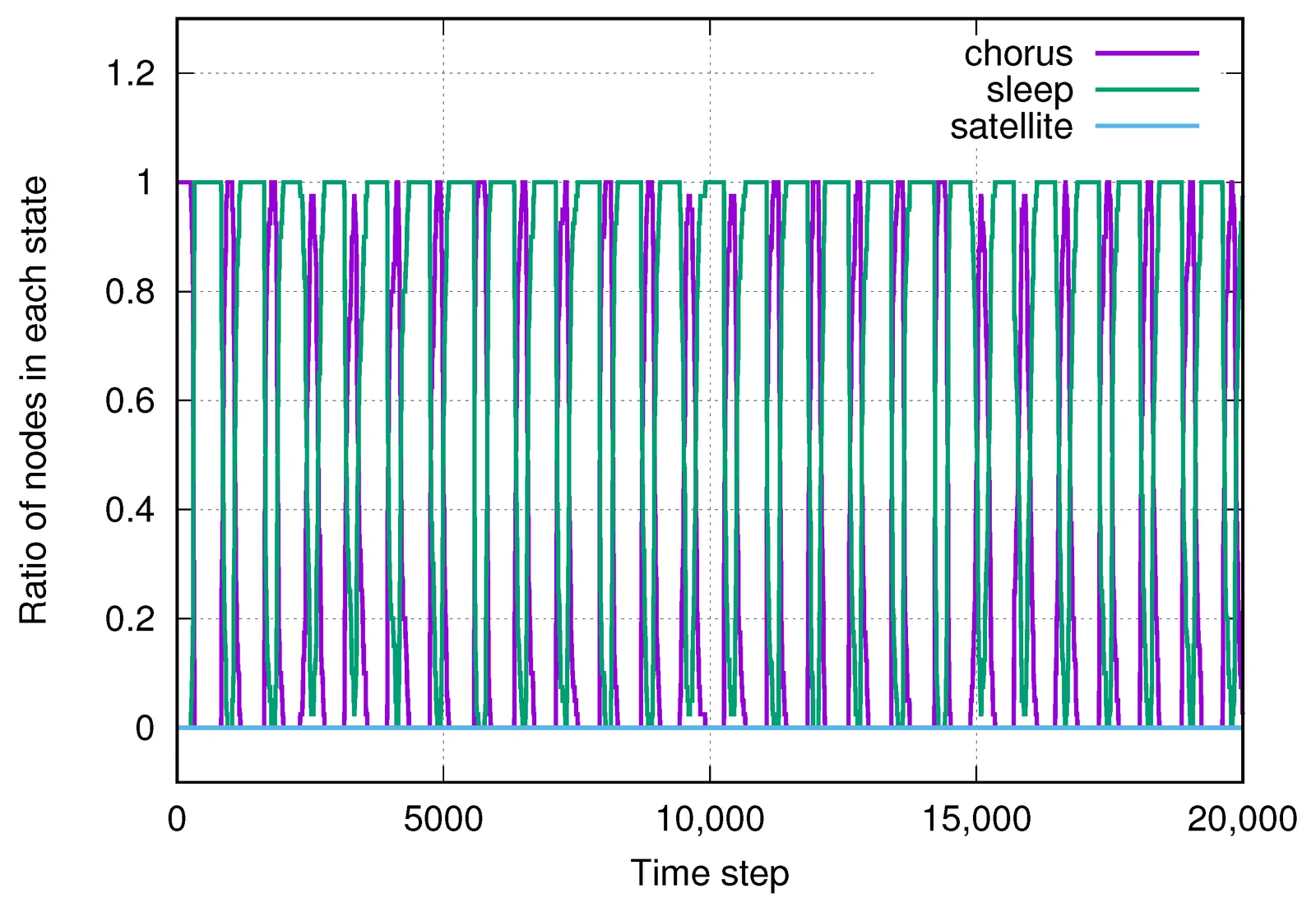
Ratio of nodes in each state without the satellite model.

**Figure 6:**
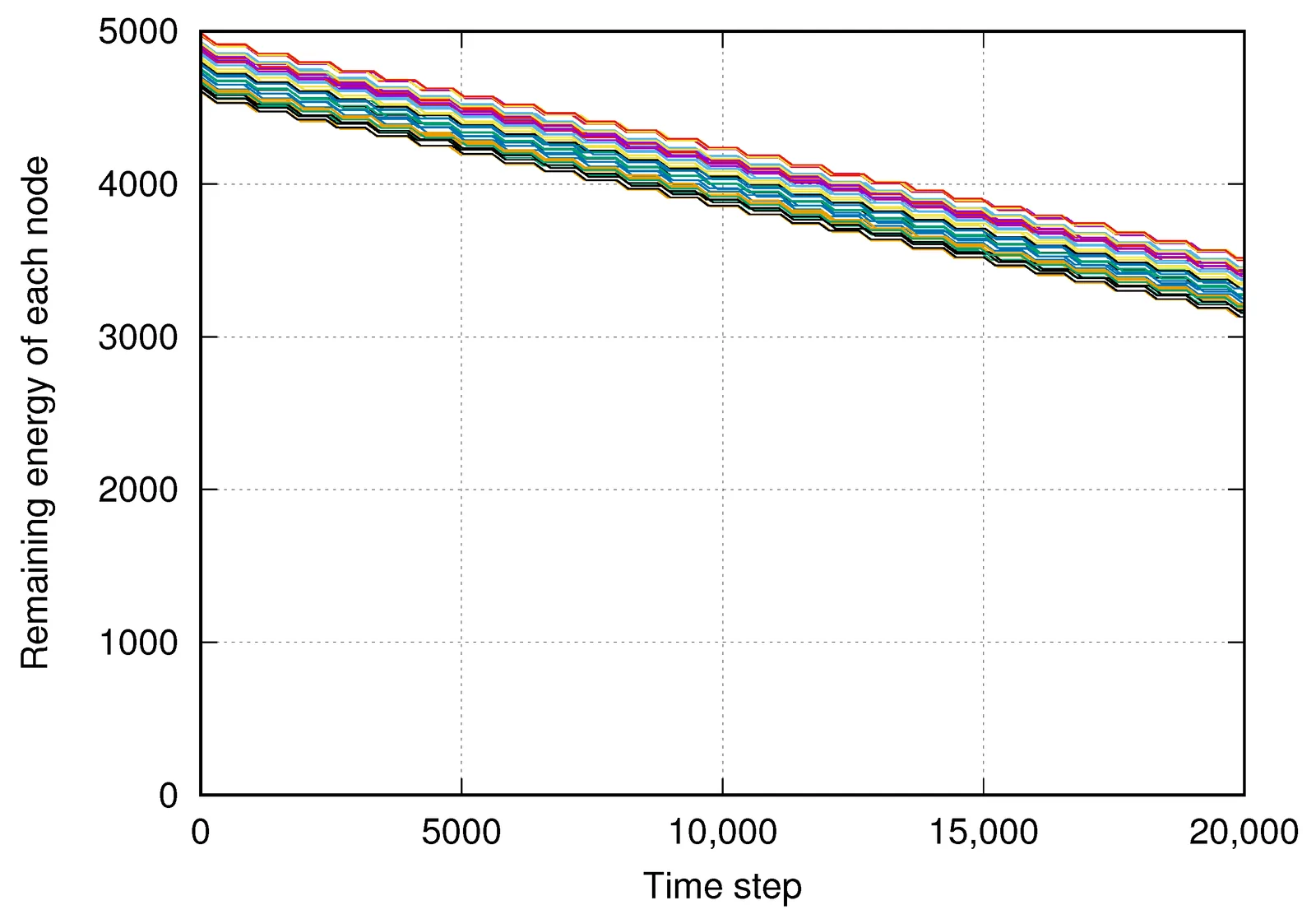
Remaining energy without the satellite model; the initial energy levels of the 40 nodes are randomly assigned.

**Figure 7:**
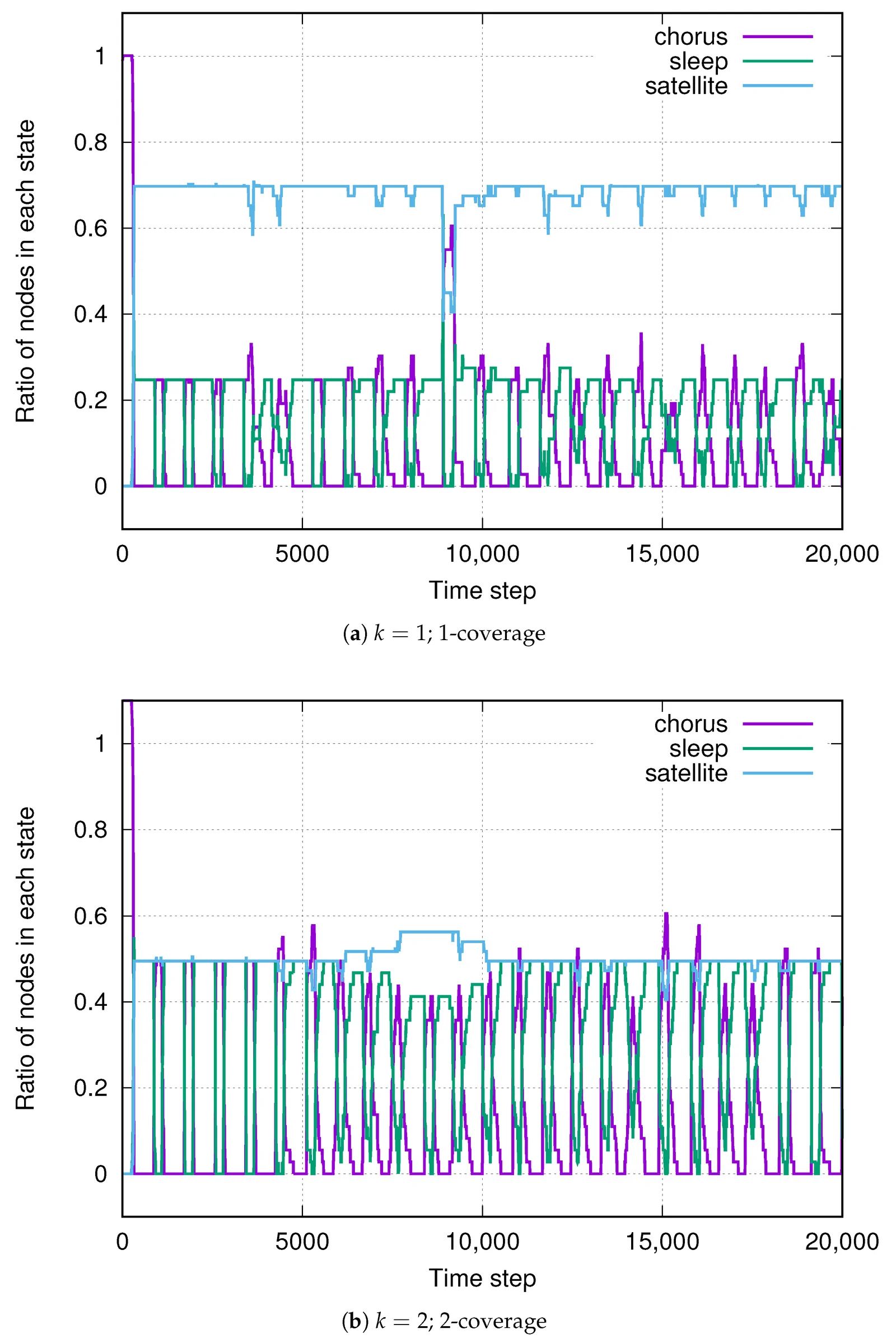
Ratio of nodes in each state.

**Figure 8:**
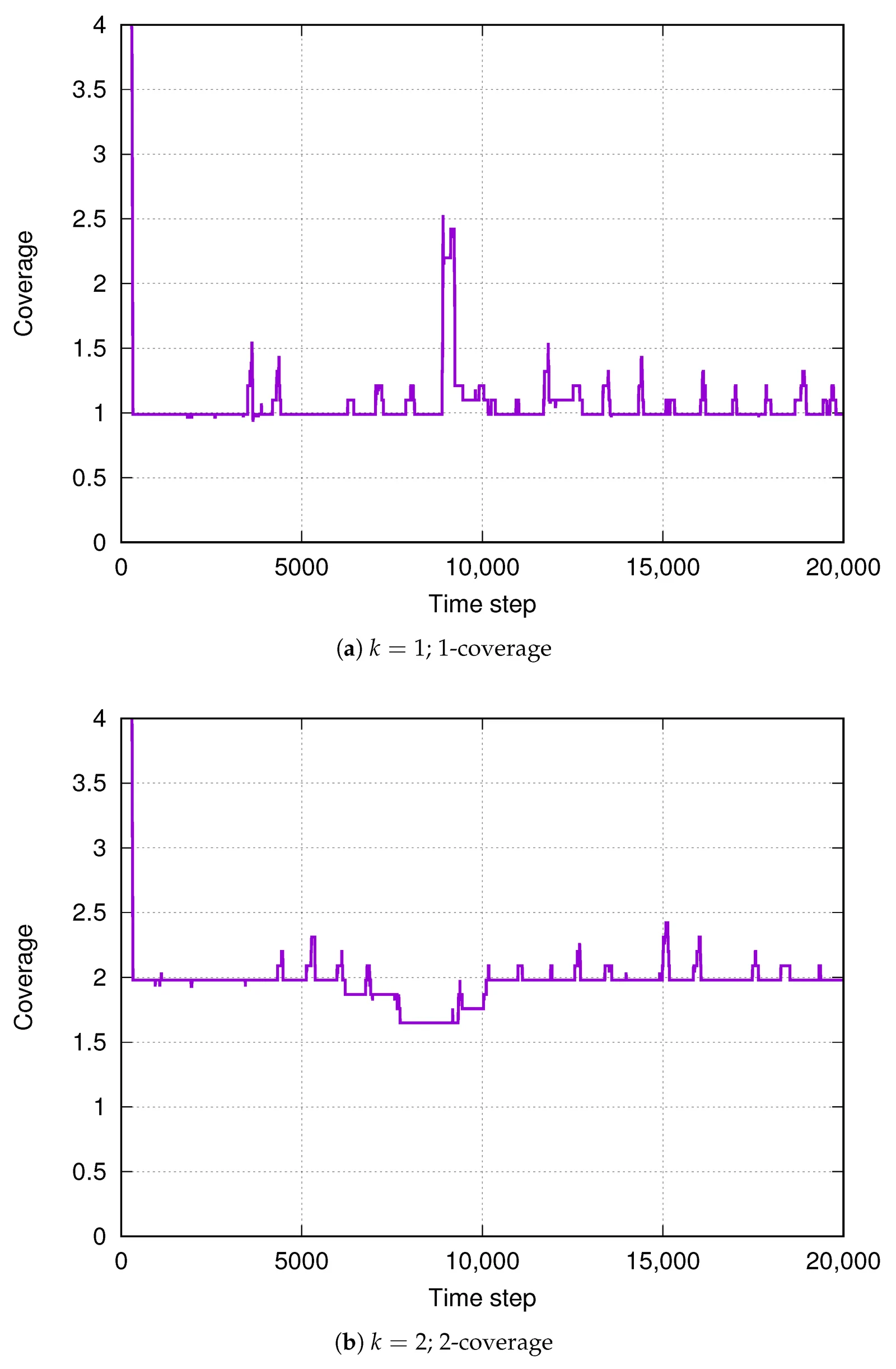
Average coverage.

**Figure 9:**
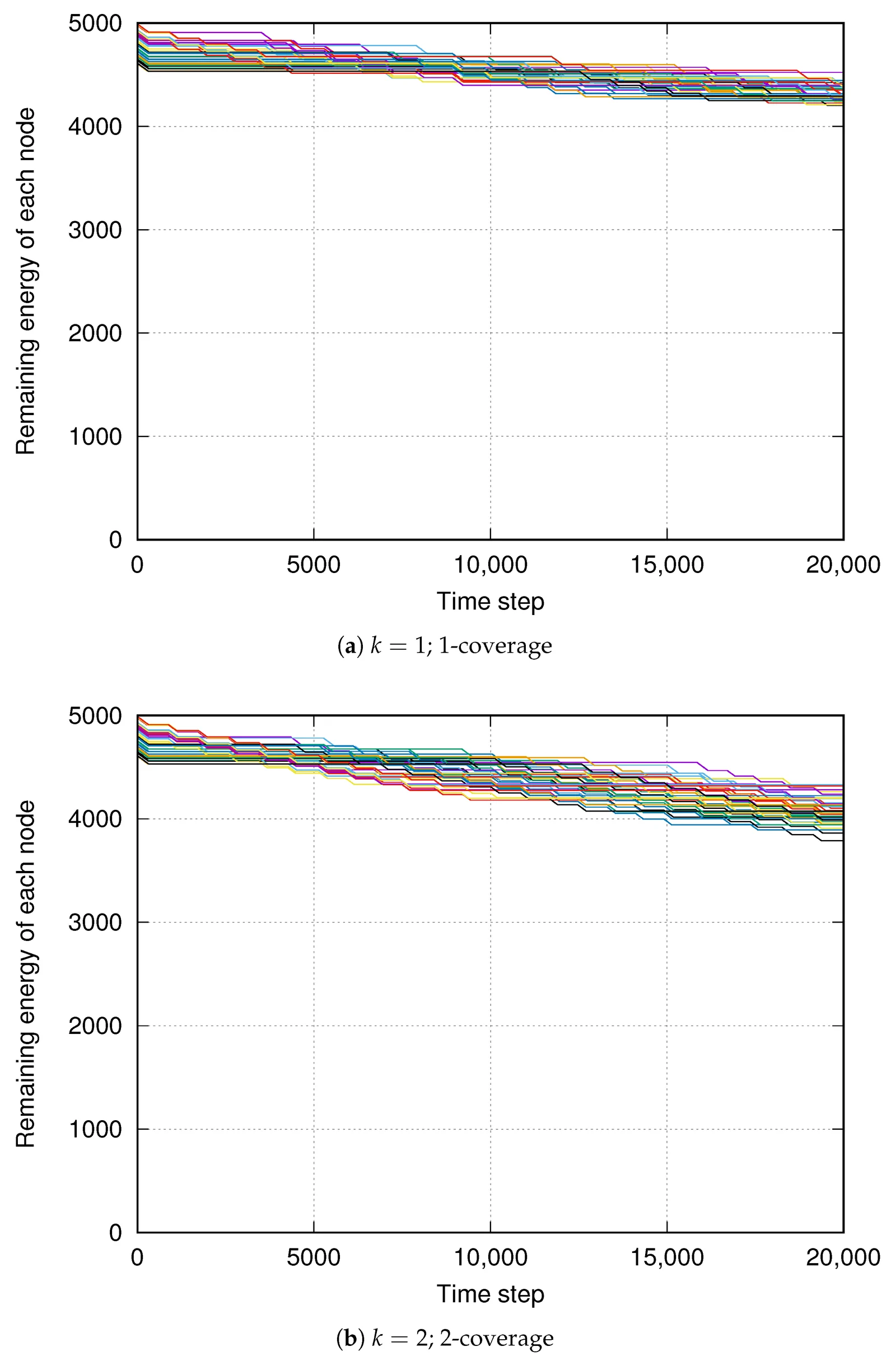
Remaining energy with the satellite model; the initial energy levels of the 40 nodes are randomly assigned. The introduction of the satellite model results in a more gradual decrease in residual energy.

**Figure 10:**
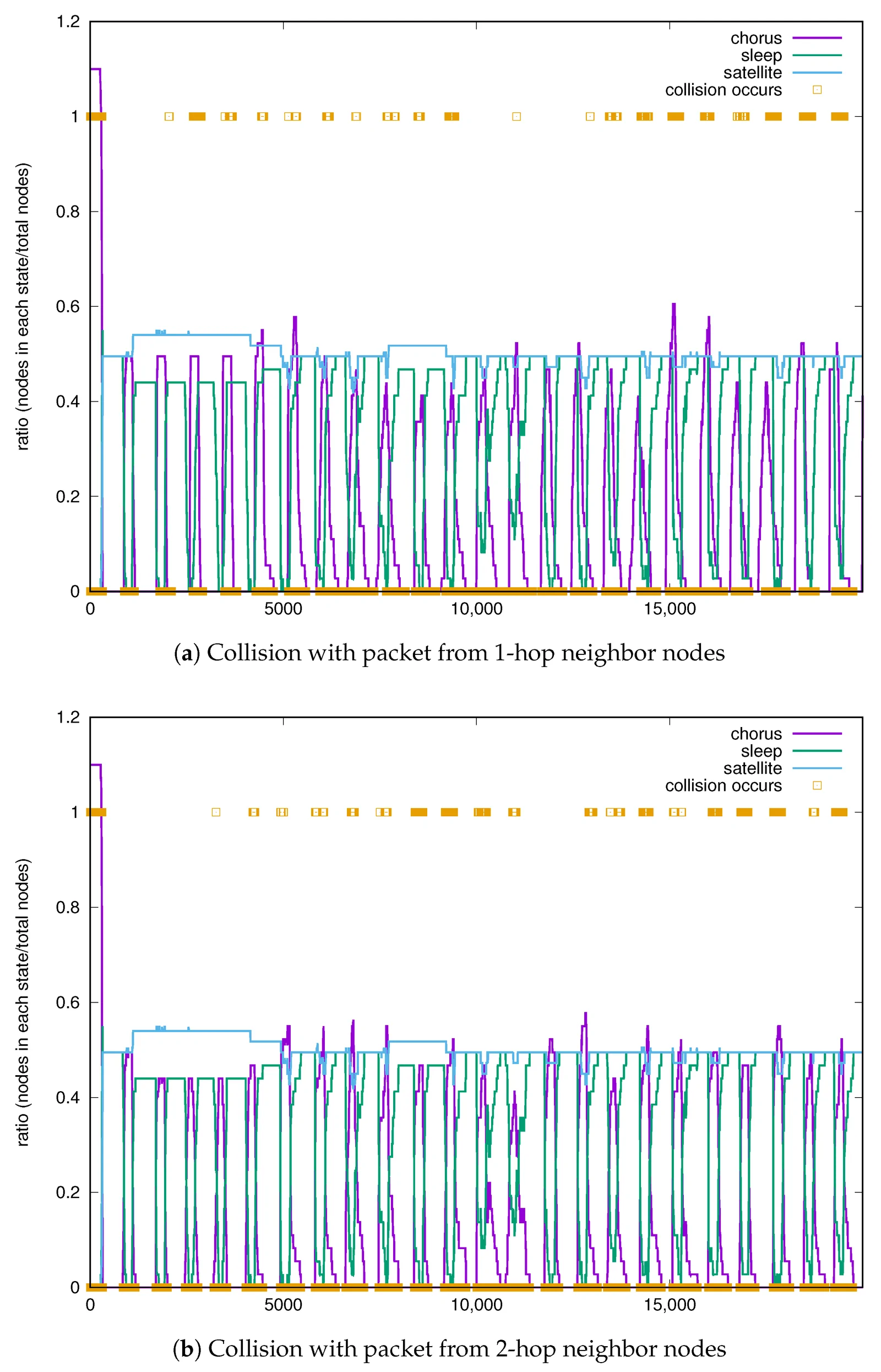
Packet collisions.

**Table 1:** Parameter interpretation of the two models.

Variables	Frog-Behavior Model	Wireless Communication
*T*	Fatigue; it limits continuous action	Air-time limitation; it limits the operation time
*E*	Physical strength	Residual power
$r_{0}$	Maximum distance between the interacting individuals	Communication range
$r_{0}^{sate}$	Maximum distance to individuals to compare attractiveness	Sensing distance

**Table 2:** Parameter settings.

Parameter	Value	Description
κ	0.01	Used in Equation (3)
α	1	Used in Equation (3)
$\alpha_{T}$	0.40	Unit of fatigue recovery
$T_{max}$	100	Maximum fatigue degree
ΔT	80	Inflection point of the sigmoid function ($G_{1},\;G_{2}$)
γ	0.5	Slope of the sigmoid function ($G_{1},\;G_{2}$)
$p_{high}$	0.8	Used in Equation (16)
$p_{low}$	0.01	Used in Equation (16)
$\lambda_{\mathrm{in}}$	10	Used in Equation (18)
$\lambda_{\mathrm{out}}$	200	Used in Equation (19)
ϵ	5	Slope of the sigmoid function ($I_{1},\;I_{2}$)

**Table 3:** Collision evaluation for the proposed phase-control mechanism. $N_{f}$ denotes the number of consecutive in-phase flags required to update the interaction parameter *S*, and $W_{f}$ denotes the maximum allowable interval between successive flags in oscillator cycles. $N_{f}=W_{f}=\infty$ represents the case where the in-phase-flag-based hidden-node avoidance mechanism is disabled. Each collision entry shows the number of collided transmissions followed by the collision rate [%] in parentheses.

Method	$N_{f}$	$W_{f}$	TX	1-hop	2-hop
Random fixed phase	–	–	30,000	1977.6 (6.592)	2807.3 (9.358)
Proposed	*∞*	*∞*	15,984	90 (0.563)	717 (4.486)
Proposed	1	1	15,312	36 (0.235)	538 (3.514)
Proposed	1	3	15,312	36 (0.235)	538 (3.514)
Proposed	1	5	15,312	36 (0.235)	538 (3.514)
Proposed	3	1	15,886	14 (0.088)	1137 (7.157)
Proposed	3	3	15,509	49 (0.316)	773 (4.984)
Proposed	3	5	15,509	49 (0.316)	773 (4.984)
Proposed	5	1	15,294	52 (0.340)	817 (5.342)
Proposed	5	3	16,067	66 (0.411)	597 (3.716)
Proposed	5	5	16,067	66 (0.411)	597 (3.716)

## Data Availability

The simulation data and source code supporting the findings of this study are available from the corresponding author upon reasonable request.
